# Islet Dimensions and Its Impact on the Cellular Composition and Insulin-Secreting Capacity: Insights Into the Role of Non-beta Cells

**DOI:** 10.7759/cureus.52428

**Published:** 2024-01-17

**Authors:** Pravash Mishra, Abhijit Sahu, Pradeep K Naik, Praveen Kumar Ravi

**Affiliations:** 1 Anatomy, All India Institute of Medical Sciences, Bhubaneswar, IND; 2 Biotechnology and Bioinformatics, Centre of Excellence in Natural Products and Therapeutics, Sambalpur University, Burla, IND

**Keywords:** non-beta cells, non-alpha cells, islets of langerhans, beta cells, insulin secretion, diabetes mellitus, india, imagej, immunohistochemistry

## Abstract

Studies have underscored the significance of islet dimensions, encompassing i) the necessity for islets to maintain an optimal diameter to sustain functional activity; ii) larger islets exhibit an intermingled architecture of alpha and beta cells, enhancing functional activity through paracrine effects; iii) non-alpha/beta (NAB) cells play a significant role in regulating beta cells; and iv) there is a preferential loss of larger islets in cases of type 2 diabetes mellitus.

To delve deeper into these aspects, the authors documented the cellular composition in islets of various dimensions and regions of the pancreas, along with their secreting capacity, using the expression of the myosin Va motor protein in nine non-diabetic adult human pancreases. The proportion of NAB cells was found to be higher in intermediate islets and significantly lower in smaller and larger islets.

By comparing the differences in islet composition, where NAB cells increase from smaller to intermediate islets, leading to a decrease in the proportion of alpha and beta cells, and in larger islets, there is a higher proportion of beta and alpha cells similar to smaller islets, we propose the hypothesis that NAB cells proliferate as islets increase in size. Furthermore, in larger islets, these NAB cells convert into alpha and beta cells, resulting in the scattered, intermingled arrangement observed in larger islets.

The higher intensity of myosin Va expression in the islets of the tail region, along with a similar proportion of NAB cells in intermediate islets of the tail region compared to larger islets, leads to decreased inhibitory stimuli to beta cells and an increased insulin-secreting capacity.

## Introduction

Diabetes mellitus (DM) is a chronic metabolic disorder characterized by impaired glucose metabolism, leading to hyperglycemia and insulin resistance. Its global impact is evident, with 422 million people affected worldwide in 2021, a number expected to rise to 700 million by 2045 [1]. In India, the prevalence of DM reached 9.3% in 2022, with approximately 57% of cases remaining undiagnosed within the community [2,3]. Particularly concerning is the burden on individuals from lower socioeconomic backgrounds, who allocate a substantial portion of their income (24-35%) to DM management once diagnosed [4,5]. The escalating prevalence of DM in developing countries underscores the need for enhanced healthcare facilities, awareness programs, and patient education to combat this growing public health issue [6].

The endocrine segment of the pancreas, comprising only 1 to 2% of its total volume [7], hosts islands of endocrine cell clusters ranging from 50 to 300 µm dispersed amid exocrine pancreatic acini [8]. With approximately 1 to 3 million islets distributed within the pancreas and a total islet volume of 0.5 to 2.0 cubic cm [9], these structures play a pivotal role in regulating glucose homeostasis throughout the body. Minor alterations in islet composition or architecture can yield significant changes in blood glucose levels by affecting the secreting capacity of various hormones [9].

Recent studies have underscored the importance of islet dimensions and their functional activity. Key considerations include i) the necessity for islets to maintain an optimal diameter for functional activity [10,11]; ii) larger islets exhibiting an intermingled architecture of alpha and beta cells, rendering them functionally more active despite a smaller proportion of beta cells, facilitated by paracrine effects [12,13]; iii) the influential role of islet dimension in determining islet composition [12,14]; iv) the crucial involvement of non-alpha/beta (NAB) cells, such as delta cells, F (pancreatic polypeptide) cells, epsilon cells, and other minor cells, in regulating beta cell insulin-secreting capacity [15-18]; v) the preferential loss of larger islets in cases of type 2 diabetes mellitus compared to non-diabetic populations [14].

NAB cells have been identified as significant influencers of islet-secreting capacity [18]. Functionality, or the secreting ability of the cells, is critical to determine and document, mainly in the human pancreas. Thus, there is a paucity of literature regarding the islet dimension and its secreting capacity. In the present study, in addition to the islet dimension and composition, we used antibodies against the myosin Va (MyoVa) motor protein to document their functionality. The myosin motor protein plays a crucial role in maintaining many biological functions, including the maturing, mobilization, maintenance, and exocytosis of the dense core vesicles in humans [19,20]. In our study, we have considered MyoVa out of all the myosin classes, as this protein is highly expressed in the endocrine cell region and is also directly involved in the localization and secretion of the endocrine granules, especially in neurons and neuroendocrine cells [19,20]. The primary objective of this study is to document the cellular composition (proportion of alpha cells, beta cells, and NAB cells) in islets of various dimensions and regions of the pancreas, along with their secreting capacity, employing the expression of the MyoVa motor protein in the non-diabetic Indian population.

## Materials and methods

Specimen collection

The study was conducted at All India Institute of Medical Sciences, Bhubaneswar, Odisha, India. A total of nine adult human non-diabetic pancreases from the Indian population were procured through autopsies of road traffic accident cases, following approval from the institutional ethical committee (approval No. T/EMF/Anatomy/19/31) and obtaining informed written consent. A comprehensive medical history, including diabetic status and comorbid conditions, was meticulously documented. An autopsy, which was done within nine hours of death, was included in the study. The specimen that showed pathological features and autolytic changes in either gross morphometry or histology was excluded from the study. Cases with diabetes and prolonged illnesses were deliberately excluded to ensure the integrity of the study results.

After conducting morphometric measurements, the collected pancreatic specimens underwent immersion fixation for 24 hours in 10% neutral buffered formalin. The pancreas was anatomically divided into head, body, and tail segments, adhering to established literature references [13,21,22]. To capture regional variations, comprehensive representative sections (coronal and axial) were obtained from the head, body, and tail regions, further subdivided into subblocks. Subsequently, the pancreatic blocks underwent processing and embedding into paraffin blocks for subsequent analyses.

Immunohistochemistry

Serial sections of 4µm thickness were prepared from each paraffin block (~40 blocks per pancreas). Immunohistochemistry (IHC) was conducted on four consecutive serial sections following heat antigen retrieval using citrate buffer under pressure. The sections underwent staining using the following rabbit monoclonal antibodies: anti-synaptophysin (1:300) from PathnSitu, Livermore, California; anti-insulin (1:200) from PathnSitu, Livermore, California; anti-glucagon (1:200) from Cell Signaling, Massachusetts, United States; and anti-myosin Va (1:300) from Sigma-Aldrich, Missouri, United States. Incubation for 45 minutes was carried out to identify the islet area, beta cell area, alpha cell area, and myosin Va motor protein within the pancreatic islets. Detection of the primary antibodies was accomplished using a secondary antibody labeled with horseradish peroxidase and DAB (3,3-diaminobenzidine) chromogen from PathnSitu, Livermore, California. Whole slide images were captured at 10x magnification and stored for subsequent analysis.

Quantification

Analysis of all whole slide images (WSIs) was performed using the Fiji/ImageJ free software from NIH (download from http://imagej.nih.gov/). For each pancreatic block, whole slide images of all four IHC stainings were concurrently examined. Three islets were selected based on diameter (<60µm - small; 100-150µm - medium; 200-250µm - large) from each block. Corresponding islets were identified in all four IHC-stained slides.

In each islet, measurements of total islet area, alpha-cell area, and beta-cell area were conducted. These values were then utilized to calculate the alpha-cell area percentage, beta-cell area percentage, and NAB (non-alpha/beta) area percentage for smaller, medium, and larger islets, employing a formula akin to previous studies [13]. Myosin Va intensity was calculated in the alpha and beta cells of smaller and larger islets. The average myosin Va intensity was computed for the alpha and beta cells of each islet. The collected data were tabulated, and the mean average for the head, body, and tail regions was calculated.

Statistical analysis

The data were summarized and presented as the mean ± SD. To compare beta-cell area, alpha-cell area, NAB area, and myosin Va intensity, a paired t-test was employed. A significance level of P < 0.05 was considered statistically significant. All statistical analyses were conducted using IBM SPSS Statistics for Windows, Version 25 (released 2017; IBM Corp., Armonk, New York, United States), and graphical representations were generated using Microsoft Excel 2019 software (Microsoft Corporation, Redmond, Washington, United States).

## Results

The mean age and BMI of the subjects were 32.56 ± 9.18 years and 21.48 ± 3.54 kg/m2, respectively.

The proportion of alpha and beta cells in islets of variable size

**Figure 1:**
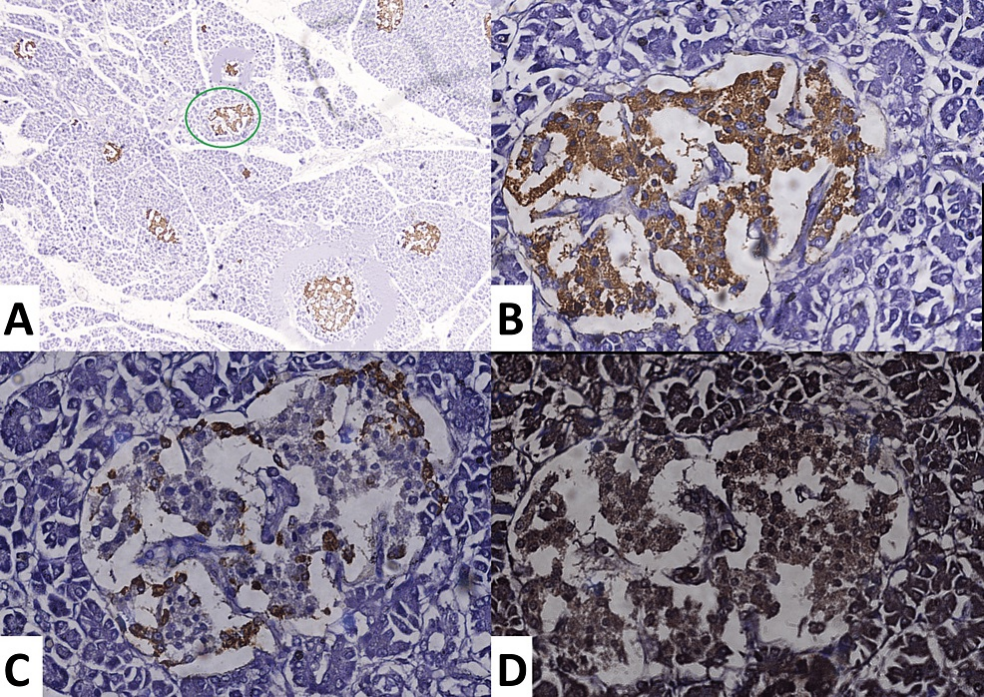
A) Lower magnification of the IHC slide stained with anti-synaptophysin antibody, showing a larger islet (green circle). B) Showing the insulin-positive area (beta cells) at higher magnification. C) Showing the glucagon-positive area (alpha cells) of the same islet. D) Showing the myosin-positive area in the next serial section. IHC: immunohistochemistry

The regional variation of alpha and beta cells in larger, intermediate, and smaller islets is tabulated in Table 1. In smaller and medium-sized islets, alpha cells were primarily situated at the periphery. Conversely, in larger islets, alpha cells were observed scattered throughout the islets, in addition to their peripheral presence (Figure 1). Beta cells predominantly occupied the central region, irrespective of the islet size. The regional variation of alpha and beta cell proportions of various islet dimensions is tabulated in Table 1.

**Table 1:** Regional variation of alpha and beta cells in islets of variable size. A%: percentage of alpha cells; B%: percentage of beta cells. * - p = 0.041; # - p < 0.0001; @ - p = 0.004.

Region	Cell	Larger islet (%)	Intermediate islet (%)	Smaller islet (%)
		Mean ± SD	Mean ± SD	Mean ± SD
Head	A%	24.61±6.83	23.11±7.09	23.91±9.87*
	B%	58.04±7.86	56.73±8.27	61.42±10.9
Body	A%	25.25±8.39	25.31±10.89	25.2±9.02
	B%	59.31±11.83	54.1±9.73	59.14±11.79
Tail	A%	25.05±5.92	24.95±7.18	26.9±8.27*
	B%	56.33±8.39	56.31±8.62	57.96±9.12
Overall	A%	25.24±7.17	24.36±8.61	25.3±8.98
	B%	57.83±9.73^@^	55.49±8.95^#^	59.44±10.61^@#^

The mean alpha cell proportions of the larger, intermediate, and smaller islets were 25.24 ± 7.17%, 24.36 ± 8.61%, and 25.3 ± 8.98%, respectively. The smaller islets of the tail region (26.9 ± 8.27) were found to have a higher proportion of alpha cells than the head region (23.91 ± 9.87%) (p= 0.041). The mean beta cell proportions of the larger, intermediate, and smaller islets were 57.83 ± 9.73%, 55.49 ± 8.95%, and 59.44 ± 10.61%, respectively. Smaller islets were found to have higher levels of beta cells (59.44 ± 10.61%) when compared with larger and intermediate islets (p = 0.0001).

The proportion of non-alpha /beta cells (NAB) in islets of variable size

Regional variations of NAB cells are detailed in Table 2. The mean NAB proportion in smaller, intermediate, and larger islets was 16.92 ± 10.36%, 20.15 ± 9.98%, and 15.25 ± 9.87%, respectively. Notably, intermediate islets exhibited a significantly higher proportion of NAB cells compared to smaller (P=0.0001) and larger islets (P=0.001). There is no regional variation in the NAB proportion among islets of similar size.

**Table 2:** Regional variation of non-alpha/beta (NAB) cells in islets of variable size. Li: larger islets; Ii: intermediate islets; Si: smaller islets

Region	Larger islet (Li)	Intermediate islet (Ii)	Smaller islet (Si)	P Value
	Mean ± SD (%)	Mean ± SD (%)	Mean ± SD (%)	
Head	17.35 ± 9.38	20.16 ± 8.65	14.67 ± 9.0	Li vs Ii – 0.04* ; Ii vs Si – 0.0001*; Li vs Si – 0.77
Body	15.10 ± 10.48	21.34 ± 12.01	15.87 ± 11.2	Li vs Ii – 0.001*; Ii vs Si – 0.003*; Li vs Si – 0.612
Tail	18.62 ± 10.95	18.74 ± 8.47	15.13 ± 9.12	Li vs Ii – 0.943; Ii vs Si – 0.024*; Li vs Si – 0.022*
Overall	16.92 ± 10.36	20.15 ± 9.98	15.25 ± 9.87	Li vs Ii – 0.001*; Ii vs Si – 0.0001*; Li vs Si – 0.057

Intensity calculation of MyoVa motor protein in endocrine cells

Regional variations in the expression of MyoVa in alpha and beta cells were documented in Table 3. The intensity of MyoVa in beta cells consistently surpassed that in alpha cells, observed in both larger and smaller islets across all regions of the pancreas, with a p-value < 0.0001. Larger islets in the tail region exhibited a significantly higher intensity of MyoVa expression in both alpha and beta cells compared to the body region, with p-values of 0.043 and 0.006, respectively. While a similar trend was observed in smaller islets, it did not reach statistical significance. Consequently, the distribution of MyoVa in both alpha and beta cells of the larger islets in the tail region was notably higher than in other regions and smaller islets.

**Table 3:** Regional variation of myosin Va expression in alpha and beta cells. * - p = 0.043; # - p = 0.006.

Region	Cell	Larger islet	Smaller islet
		Mean ± SD (%)	Mean ± SD (%)
Head	Alpha cell	94.35 ± 12.38	92.56 ± 16.61
	Beta cell	97.98 ± 10.22	98.53 ± 17.39
Body	Alpha cell	91.06 ± 13.55*	94.07 ± 13.93
	Beta cell	94.01 ± 12.48^#^	98.96 ± 17.29
Tail	Alpha cell	95.03 ± 15.26*	96.02 ± 14.22
	Beta cell	100.95 ± 15.6^#^	101.42 ± 12.5
Overall	Alpha cell	93.77 ± 14.17	94.2 ± 15.7
	Beta cell	98 ± 13.59	99.2 ± 16.64

## Discussion

The current study extensively explores the distribution of alpha, beta, and NAB cells within islets of various dimensions, coupled with an analysis of MyoVa expression. Islet dimension exhibits a broad spectrum, ranging from small cell clusters to larger islets comprising hundreds of cells. Quantifying islets in the human pancreas is inherently challenging due to their intricate distribution. Large-scale computer-assisted analyses are employed by researchers to mitigate bias and emphasize the pivotal role of the islet dimension in islet composition and insulin-secreting capacity [23-25]. Analyzing the impact of the islet dimension on its composition becomes challenging when quantifying an unequal number of islets in each group. Considering the diverse distribution of islets in the pancreatic tissue as a whole, achieving an equal number of islets in the small, medium, and larger islet groups based on their dimensions is improbable. Therefore, the current study adopts the approach of documenting the proportion of alpha and beta cells within a fixed number of small, medium, and large islets. This methodological choice enhances the precision of our findings and contributes to a more accurate understanding of the intricate relationship between islet dimensions, cellular composition, and insulin-secreting capacity.

Comparative studies on islets have consistently reported that the size of islets remains relatively constant regardless of organism size, whether in rodents, primates, or humans, staying within specific limits [10,12]. This observation led the authors to formulate the hypothesis that maintaining an optimal islet size is crucial for ensuring its functional activity [10,12]. Even minor changes in islet dimension can lead to significant variations in cellular arrangement and composition, thereby influencing insulin-secreting capacity through paracrine effects [18,26]. Recent studies highlighted a reduction in the population of larger islets in the pancreas of individuals with type 2 diabetes mellitus (T2DM) compared to their non-diabetic, healthy counterparts, underscoring the pivotal role of the islet dimension in diabetes pathology [25]. This reinforces the significance of investigating islet dimensions in understanding the pathophysiology of diabetes and its potential implications for therapeutic interventions.

**Figure 2:**
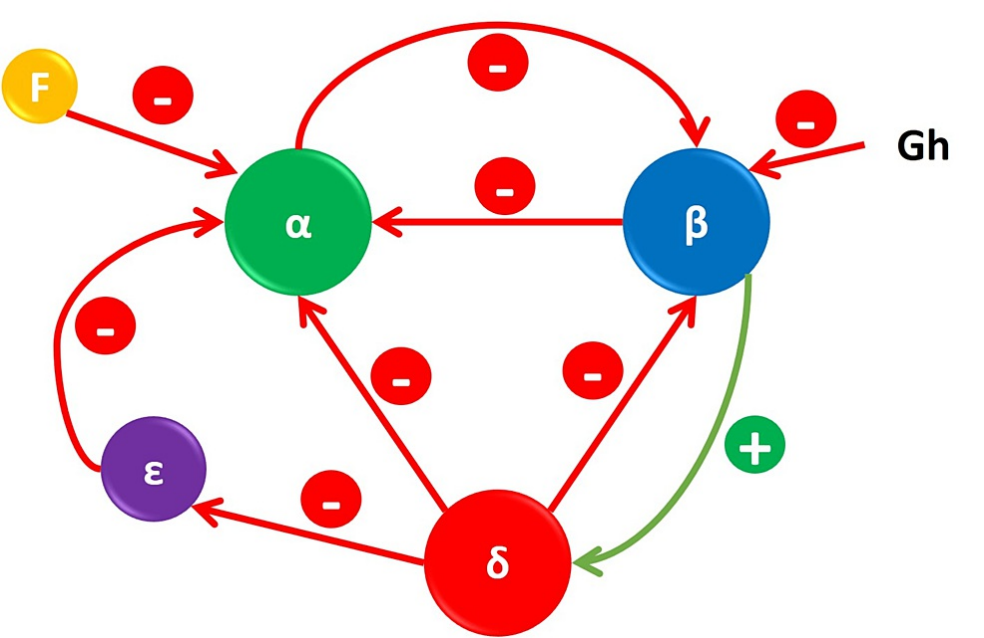
The paracrine influence of beta cells by non-beta cells. Image credits: Praveen Kumar Ravi

We observed an increase in the proportion of beta cells in smaller islets, aligning with findings from prior studies [13]. Smaller islets in the tail region exhibited a higher proportion of alpha cells compared to the head and body regions of the pancreas. Additionally, the proportion of non-alpha/beta (NAB) cells was higher in intermediate islets and significantly lower in both smaller and larger islets. Islets, functioning as small micro-organs, coordinate their activities in response to various stimuli, including glucose concentration, insulin, glucagon, somatostatin, ghrelin, pancreatic polypeptide, and various neurotransmitters [18]. While alpha and beta cells have been extensively studied, NAB cells remain less explored. NAB cells play a crucial role in regulating beta cell function through paracrine effects and contribute significantly to replenishing beta cell mass by converting non-beta cells into beta cells [27-30]. The intricate paracrine interactions between beta and non-beta cells are depicted in Figure 2 [18,30]. Most non-beta cells inhibit beta cell secretion, and a reduction in the proportion of non-beta cells might be advantageous for the insulin-secreting capacity of islets.

**Figure 3:**
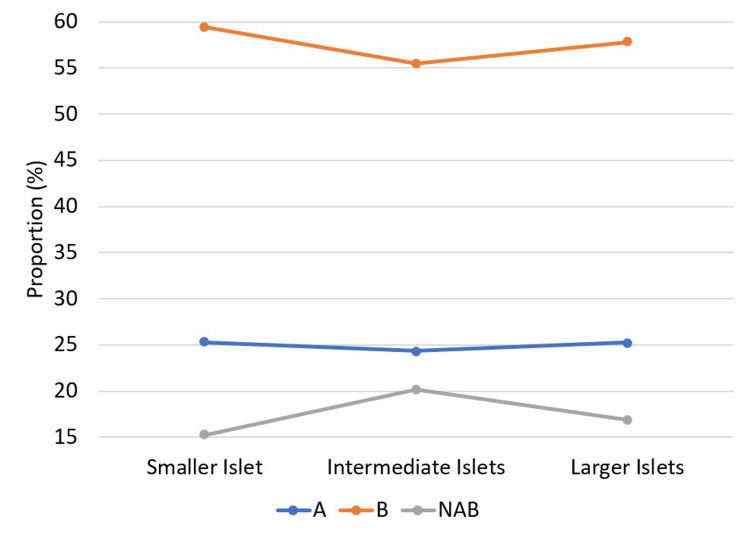
Changes in the islet composition in relation to its dimension. A: alpha cell; B: beta cell; NAB: non-alpha/beta cell. This figure has been created by the authors by using the data from the present study.

Numerous studies in animal models have documented an increase in islet dimension and the conversion of non-beta cells to beta cells in response to hyperglycemia [10,12,28,29]. Comparing the composition of islets in smaller, intermediate, and larger sizes in the current study (Figure 3), where NAB cells increase from smaller to intermediate islets, leading to a decrease in the proportion of alpha and beta cells, and in larger islets, there is a higher proportion of beta and alpha cells similar to smaller islets, we propose the hypothesis that NAB cells proliferate as the islet increases in size. Furthermore, in larger islets, these NAB cells convert into alpha and beta cells, providing a plausible explanation for the scattered, intermingled arrangement observed in larger islets. This hypothesis contributes to our understanding of the dynamic cellular changes within islets of varying sizes.

Studying the insulin-secreting capacity of islets through glucose-induced insulin secretion is typically performed in in vivo models [31,32]. However, conducting such experiments in the human pancreas is both expensive and challenging. In our study, we opted to quantify the expression of MyoVa as a surrogate marker to depict the functional activity of islets. Numerous studies have highlighted the involvement of MyoVa in the transport of various granules, particularly insulin secretory granules in pancreatic beta cells, secretory vesicles in chromaffin cells, pigment granules in melanocytes, and the smooth endoplasmic reticulum in neurons [20,33,34]. In diabetic models induced by streptozotocin, there was a reported decrease in the expression of MyoVa (both protein and mRNA) and MyoIIB in brain tissue compared to non-diabetic controls [35,36]. This observation suggests a potential dysfunction or downregulation of MyoVa in diabetes, impacting the movement of organelles or secretory vesicles. Hyperglycemia is believed to stimulate the expression of MyoVa, contributing to insulin secretion. The failure of this stimulatory mechanism might be one of the multifactorial contributors to the development of diabetes mellitus (DM) [37]. Our utilization of MyoVa expression provides valuable insights into the functional status of islets and their potential role in the context of diabetes.

Islets in the tail region are recognized for their larger size and superior insulin-secreting capacity, as reported in previous studies [11,14]. Our observation of higher MyoVa expression intensity in the islets of the tail region supports this notion. Furthermore, NAB cells in intermediate islets of the tail region exhibit similarities to those in larger islets, contributing to a reduction in inhibitory stimuli to beta cells and consequently an increase in insulin-secreting capacity.

The present study is done on a single paraffin section; it might be one of the limitations, as we may consider the terminal part of larger islets as smaller islets. This limitation is common in many studies in the literature, as none, to the best of our knowledge, has been conducted on serial sections to identify the maximum dimension of islets for categorization as larger or smaller. We recognize the potential for underestimating the islet area, but we believe this limitation is minimized in our study due to the analysis of a substantial number of islets. Nonetheless, considering this limitation, future studies incorporating serial sections could provide a more comprehensive understanding of islet dimensions and their functional implications.

## Conclusions

In conclusion, our study provides valuable insights into the intricate relationship between islet dimensions, cellular composition, and insulin-secreting capacity in the human pancreas. We observed notable variations in the proportions of alpha, beta, and non-alpha/beta (NAB) cells across islets of different sizes, with smaller islets demonstrating increased beta cell proportions and tail region islets exhibiting elevated alpha cell proportions. The evolving understanding of type 2 diabetes mellitus (T2DM) as a bi-hormonal disorder involving both insulin and glucagon suggests the potential characterization of T2DM as a multi-hormonal disorder with further exploration into the role of NAB cells.

As we continue to unravel the complexities of islet biology, the insights gained from this study regarding the islet dimension and its impact on cellular composition and insulin-secreting capacity hold promise for practical applications. Specifically, this knowledge could be leveraged during islet harvesting and transplantation processes, aiming to enhance success rates. The recognition of islet heterogeneity and the nuanced interplay between different cell types within islets pave the way for more targeted approaches in diabetes research and therapeutic interventions.

Future studies, incorporating serial sections for a more detailed assessment of islet dimensions, could further enhance our understanding of the dynamic changes within islets and their implications for diabetes pathology. Overall, our findings contribute to the ongoing discourse on the significance of islet heterogeneity and its impact on pancreatic function.

## References

[1] (2023). World Health Organization: Global report on diabetes. https://apps.who.int/iris/handle/10665/204871.

[2] Mathur P, Leburu S, Kulothungan V (2022). Prevalence, awareness, treatment and control of diabetes in India from the Countrywide national NCD monitoring survey. Front Public Health.

[3] Pradeepa R, Mohan V (2021). Epidemiology of type 2 diabetes in India. Indian J Ophthalmol.

[4] Leon BM, Maddox TM (2015). Diabetes and cardiovascular disease: epidemiology, biological mechanisms, treatment recommendations and future research. World J Diabetes.

[5] Pandey A, Chawla S, Guchhait P (2015). Type-2 diabetes: current understanding and future perspectives. IUBMB Life.

[6] Grant P (2013). Management of diabetes in resource-poor settings. Clin Med (Lond).

[7] Schoenwolf GC, Bleyl S, Brauer PR, Francis-West PH (2009). Larsen’s Human Embryology. https://shop.elsevier.com/books/larsens-human-embryology/schoenwolf/978-0-323-69604-3.

[8] Ross MH, Pawlina W (2011). Histology: A Text and Atlas: With Correlated Cell and Molecular Biology. https://books.google.co.in/books/about/Histology.html?id=JlxqAAAAMAAJ&redir_esc=y.

[9] Ionescu-Tirgoviste C, Gagniuc PA, Gubceac E, Mardare L, Popescu I, Dima S, Militaru M (2015). A 3D map of the islet routes throughout the healthy human pancreas. Sci Rep.

[10] Kilimnik G, Jo J, Periwal V, Zielinski MC, Hara M (2012). Quantification of islet size and architecture. Islets.

[11] Ravi PK, Singh SR, Mishra PR (2021). Redefining the tail of pancreas based on the islets microarchitecture and inter-islet distance: an immunohistochemical study. Medicine (Baltimore).

[12] Kim A, Miller K, Jo J, Kilimnik G, Wojcik P, Hara M (2009). Islet architecture: a comparative study. Islets.

[13] Ravi PK, Purkait S, Agrawal U, Patra S, Patnaik M, Singh SR, Mishra PR (2019). Regional variation of human pancreatic islets dimension and its impact on beta cells in Indian population. Islets.

[14] Wang X, Misawa R, Zielinski MC (2013). Regional differences in islet distribution in the human pancreas--preferential beta-cell loss in the head region in patients with type 2 diabetes. PLoS One.

[15] Striegel DA, Hara M, Periwal V (2015). The beta cell in its cluster: stochastic graphs of beta cell connectivity in the islets of Langerhans. PLoS Comput Biol.

[16] Zhu W, Tanday N, Flatt PR, Irwin N (2023). Pancreatic polypeptide revisited: potential therapeutic effects in obesity-diabetes. Peptides.

[17] Adams MT, Blum B (2022). Determinants and dynamics of pancreatic islet architecture. Islets.

[18] Da Silva Xavier G (2018). The cells of the islets of Langerhans. J Clin Med.

[19] Kögel T, Bittins CM, Rudolf R, Gerdes HH (2010). Versatile roles for myosin Va in dense core vesicle biogenesis and function. Biochem Soc Trans.

[20] Espindola FS, Banzi SR, Calabria LK (2008). Localization of myosin-Va in subpopulations of cells in rat endocrine organs. Cell Tissue Res.

[21] Ravi PK, Purkait S, Singh SR, Mishra PR (2022). Decay score: a guide to the immunoreactivity of human pancreatic islets in autopsy specimen. Folia Morphol (Warsz).

[22] Campbell-Thompson ML, Montgomery EL, Foss RM, Kolheffer KM, Phipps G, Schneider L, Atkinson MA (2012). Collection protocol for human pancreas. J Vis Exp.

[23] Poudel A, Fowler JL, Zielinski MC, Kilimnik G, Hara M (2016). Stereological analyses of the whole human pancreas. Sci Rep.

[24] Olehnik SK, Fowler JL, Avramovich G, Hara M (2017). Quantitative analysis of intra- and inter-individual variability of human beta-cell mass. Sci Rep.

[25] Kilimnik G, Zhao B, Jo J, Periwal V, Witkowski P, Misawa R, Hara M (2011). Altered islet composition and disproportionate loss of large islets in patients with type 2 diabetes. PLoS One.

[26] Unger RH, Orci L (2010). Paracrinology of islets and the paracrinopathy of diabetes. Proc Natl Acad Sci U S A.

[27] Chakravarthy H, Gu X, Enge M (2017). Converting adult pancreatic islet α cells into β cells by targeting both Dnmt1 and Arx. Cell Metab.

[28] Rodriguez UA, Socorro M, Criscimanna A (2021). Conversion of α-Cells to β-Cells in the postpartum mouse pancreas involves Lgr5 progeny. Diabetes.

[29] Spears E, Serafimidis I, Powers AC, Gavalas A (2021). Debates in pancreatic beta cell biology: proliferation versus progenitor differentiation and transdifferentiation in restoring β cell mass. Front Endocrinol (Lausanne).

[30] Guney MA, Lorberbaum DS, Sussel L (2020). Pancreatic β cell regeneration: to β or not to β. Curr Opin Physiol.

[31] Henquin JC (2021). Glucose-induced insulin secretion in isolated human islets: does it truly reflect β-cell function in vivo?. Mol Metab.

[32] Ahrén B (2022). The glucose sensitivity of insulin secretion-lessons from in vivo and in vitro studies in mice. Biomolecules.

[33] Waselle L, Coppola T, Fukuda M, Iezzi M, El-Amraoui A, Petit C, Regazzi R (2003). Involvement of the Rab27 binding protein Slac2c/MyRIP in insulin exocytosis. Mol Biol Cell.

[34] Rosé SD, Lejen T, Casaletti L, Larson RE, Pene TD, Trifaró JM (2003). Myosins II and V in chromaffin cells: myosin V is a chromaffin vesicle molecular motor involved in secretion. J Neurochem.

[35] da Costa AV, Calábria LK, Furtado FB (2013). Neuroprotective effects of Pouteria ramiflora (Mart.) Radlk (Sapotaceae) extract on the brains of rats with streptozotocin-induced diabetes. Metab Brain Dis.

[36] da Costa AV, Calábria LK, Nascimento R, Carvalho WJ, Goulart LR, Espindola FS (2011). The streptozotocin-induced rat model of diabetes mellitus evidences significant reduction of myosin-Va expression in the brain. Metab Brain Dis.

[37] Varadi A, Tsuboi T, Rutter GA (2005). Myosin Va transports dense core secretory vesicles in pancreatic MIN6 β-cells. Mol Biol Cell.

